# Targeting P-glycoprotein function, p53 and energy metabolism: Combination of metformin and 2-deoxyglucose reverses the multidrug resistance of MCF-7/Dox cells to doxorubicin

**DOI:** 10.18632/oncotarget.14373

**Published:** 2016-12-30

**Authors:** Chaojun Xue, Changyuan Wang, Yaoting Sun, Qiang Meng, Zhihao Liu, Xiaokui Huo, Pengyuan Sun, Huijun Sun, Xiaodong Ma, Xiaochi Ma, Jinyong Peng, Kexin Liu

**Affiliations:** ^1^ Department of Clinical Pharmacology, College of Pharmacy, Dalian Medical University, Dalian, China; ^2^ Department of Clinical Pharmacology, College of Pharmacy, Dalian Medical University, Dalian, China; ^3^ Department of Pharmacy, Hebei General Hospital, Shijiazhuang, China

**Keywords:** multidrug resistance, P-glycoprotein, p53, energy metabolism

## Abstract

Multidrug resistance(MDR) is a major obstacle to efficiency of breast cancer chemotherapy. We investigated whether combination of metformin and 2-deoxyglucose reverses MDR of MCF-7/Dox cells and tried to elucidate the possible mechanisms. The combination of metformin and 2-deoxyglucose selectively enhanced cytotoxicity of doxorubicin against MCF-7/Dox cells. Combination of the two drugs resumed p53 function via inhibiting overexpression of murine doubleminute 2(MDM2) and murine doubleminute 4(MDM4) leading to G2/M arrest and apoptosis in MCF-7/Dox cells. Combination of the two drugs had no effect on P-glycoprotein mRNA expression and P-glycoprotein ATPase activity but increased doxorubicin accumulation in MCF-7/Dox cells. The increased doxorubicin accumulation maybe associate with metabolic stress. Combination of metformin and 2-deoxyglucose initiated a strong metabolic stress in MCF-7/Dox cells via inhibiting glucose uptake, lactate, fatty acid, ATP production and protein kinase B(AKT)/ mammalian target of rapamycin(mTOR) pathway. Taken together, combination of metformin and 2-deoxyglucose reverses MDR of MCF-7/Dox cells by recovering p53 function and increasing doxorubicin accumulation. Furthermore, doxorubicin selectively increases MCF-7/Dox apoptosis via aggravating metabolic stress induced by metformin plus 2-deoxyglucose. The mutually reinforcing effect made the combination of metformin and 2DG had a better effect on reversing MDR.

## INTRODUCTION

Breast cancer, the most common malignancy, injures the health of women seriously. Therapies include surgery, radiotherapy and systemic treatment [1]. The goodnews is that the 5-year overall survival rate of breast cancer patients is extremely high with the development of treatment. But 30% of breast cancer will develop recurrent or metastatic disease and chemotherapy agents are limited by multidrug resistance (MDR), these problems still plague the clinical treatment of breast cancer [2].

Taxanes, paclitaxel, docetaxel and doxorubicin are the most commonly used cytotoxic drugs for breast cancer [3]. But MDR occurs after long-term use of cytotoxic drugs. The resistance of cancer cells to structurally and mechanistically unrelated classes of anticancer drugs is known as MDR [4]. The development of MDR is possibly the result of several changes in breast cancer include efflux transporter, uptake transporter, drug metabolizing enzymes, apoptotic, DNA damage repair pathway and other candidate mechanisms [5].

The most prominent mechanisms underlying MDR is overexpression of ATP-binding cassette(ABC) transporters. The most well known ABC transporters is the P-glycoprotein(P-gp) encoded by the multidrug resistance gene 1(MDR1) [6]. Another important mechanism with regard to MDR is apoptotic modulation [7]. The p53 protein regulates the cellular response to a variety of cellular stress signals. Directly or indirectly, p53 function is deregulated in numerous cancer types. p53 mutations occur in about 50% of all cancers, which directly suppress the p53 function. Overexpression of its main negative regulator murine double minute 2 (MDM2) indirectly suppress p53 function [8, 9]. Targeting p53 is effective way of anti-tomor in several cancer lines [10, 11]. The combination of metformin and 2-deoxyglucose(2DG) induces p53-dependent apoptosis in prostate cancer cells [12]. Also, p53 has shown important role in drug chemosensitivity and drug resistance [13].

Cancer cells are characterized by uncontrolled and rapid proliferation. Cancer cells typically have high levels of glucose uptake regardless of the availability of oxygen (Warburg effect) [14]. Indeed, because of the higher energy needs, cancer cells are more sensitive to changes in energy. Energy disruptors(such as biguanides, 2-deoxyglucose) obviously suppress several cancer cell proliferation [15]. MDR cancer cells frequently require more energy because ABC transporters hydrolyze ATP to transport substrates [16, 17]. ABC transporter substrates could increase metabolic cost of resistance and suppress proliferation of drug-resistance phenotypes [17]. Our prior research showed that P-gp substrate selectively increased the induced effect of combination of metformin and 2DG on apoptosis in K562/Dox cells [18].

The present study was performed to clarify whether combination of metformin and 2DG reverses MDR in MCF/Dox cells and tried to elucidate the possible molecular mechanisms.

## RESULTS

### Combination of metformin and 2DG selectively increased cytotoxicity of doxorubicin in MCF-7/Dox cells

**Figure 1:**
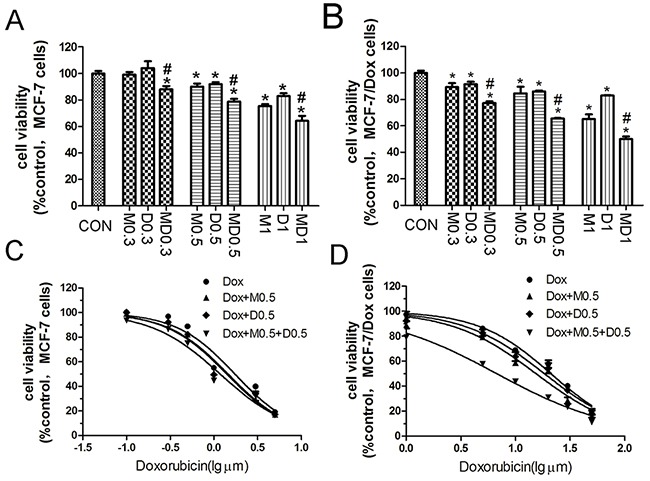
Metformin and 2-deoxyglucose(2DG) combination enhanced the cytotoxicity of doxorubicin in MCF-7/Dox cells **A, B.** MCF-7 and MCF-7/Dox cells were treated with metformin(0.3, 0.5, 1 mM) and 2DG(0.3, 0.5, 1 mM) for 24h, and the cell viability was determined by MTT assay. C, D. Cells were pretreated with indicated drugs followed by incubation with various concentrations of doxorubicin for an additional 24h. Concentrations of doxorubicin were 0, 0.10, 0.30, 0.50, 1.00, 3.00, 5.00 μM for MCF-7 cells and 0, 1, 5, 10, 20, 30, 50 μM for MCF-7/Dox cells. Columns, data are expressed as mean ± SD. *P < 0.05 Significantly different from control group. M0.5: 0.5 mM metformin, D0.5: 0.5 mM 2DG, MD0.5: 0.5 mM metformin plus 0.5 mM 2DG.

First, the cytotoxicity of metformin and 2-de-oxyglucose(2DG) against MCF-7 (Figure 1A) and MCF-7/Dox (Figure 1B) cells treated for 24h was determined by MTT assay. 0.5 mM metformin and 0.5 mM 2DG, which had cytotoxicity in both cell lines, were selected in the next MDR reversal study. Then, a possible effect of metformin or 2DG or combination of two drugs on doxorubicin cytotoxicity was examined in MCF-7 (Figure 1C) and MCF-7/Dox (Figure 1D) cells. IC50 values of doxorubicin in MCF-7 and MCF-7/Dox cells were 1.74±0.23μM and 21.12±1.89μM respectively (Table 1). MCF-7/Dox cells displayed lower cytotoxicity of doxorubicin than that of the parental MCF-7 cells. In the presence of metformin plus 2DG, IC50 values of doxorubicin in MCF-7/Dox cells markedly decreased (Table 1). Combination of metformin and 2DG selectively increased cytotoxicity of doxorubicin in MCF-7/Dox cells.

**Table 1:** IC50 values for doxorubicin of Figure 1C and Figure 1D in MCF-7 and MCF-7/Dox cells

IC50 (μM)	MCF-7	MCF-7/Dox
Dox	1.74±0.23	21.12±1.89
Dox^b^+2DG^d^	1.45±0.16	15.44±2.00
Dox^b^+MET^c^	1.40±0.16	18.41±2.30
Dox^b^+MET^c^+2DG^d^	1.29±0.12	6.80±0.70^a^

a p<0.001 vs Doxorubicin only group in MCF-7/ Dox cells.

b Dox: Doxorubicin.

c MET: metformin

d 2DG: 2-deoxyglucose.

### Combination of metformin and 2DG reversed MDR through MDM2/MDM4/p53 pathway

**Figure 2:**
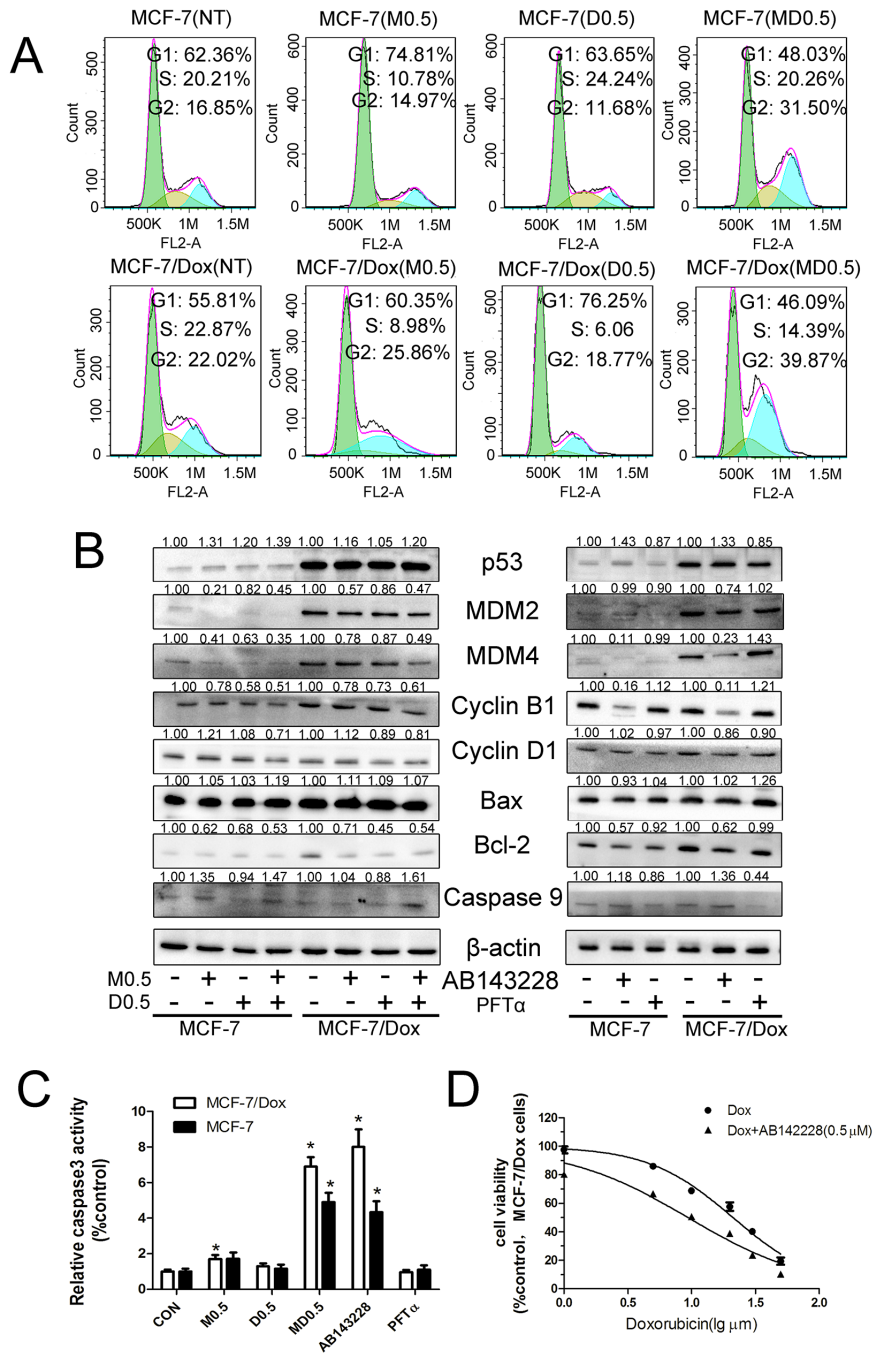
Metformin and 2-deoxyglucose(2DG) combination increased p53 expression to enhance the cytotoxicity of doxorubicin A. Cell cycle was determined by flow cytometric after treating with indicated drugs for 24h. B. Metformin 2DG combination and AB143228 increased p53 expression via inhibiting the overexpression of MDM2 and MDM4, as well as its effectors Cyclin B1, Cyclin D1, BAX, Bcl-2, caspase9. C. Metformin 2DG combination and AB143228 increased caspase3 activity. D. Cells were pretreated with AB143228(1μM) followed by incubation with various concentrations of doxorubicin(1, 5, 10, 20, 30, 50 μM) for an additional 24h. Columns, data are expressed as mean ± SD. *P < 0.05 Significantly different from control group. M0.5: 0.5 mM metformin, D0.5: 0.5 mM 2DG, MD0.5: 0.5 mM metformin plus 0.5 mM 2DG.

In order to understand the reason that combination of metformin and 2DG reversed MDR in MCF/Dox cells, we detected the effects of metformin and 2DG on cell cycle and caspase3. Metformin or 2DG induced a cell cycle arrest in G0-G1, but combination of metformin and 2DG blocked cell cycle in G2-M in both cell lines (Figure 2A). Metformin or 2DG had no effect on caspase3 activity, but combination of two drugs significantly increased caspase3 activity in both cell lines (Figure 2C). Since the p53 is the key protein to regulate the cell cycle and apoptosis, p53 and related proteins were detected by western blot. Compared with MCF-7, expressions of p53, MDM2 and MDM4 were significantly higher in MCF/Dox cells. The combination of metformin and 2DG intensively reduced expressions of MDM2 and MDM4 in both cell lines. Furthermore, combination of two drugs reduced expressions of Cyclin B1 and Cyclin D1, mildly increased BAX expression and significantly reduced Bcl-2 expression (Figure 2B). The increased proportion of BAX/ Bcl-2 enhanced apoptosis. Combination of the two drugs increased caspase9 expression (Figure 2B) and caspase3 activity (Figure 2C). Meanwhile, p53 activator AB143228 was detected as positive control, p53 inhibitor PFTα was detected as negative control. AB143228 blocked MDM4 expression and increased p53 expression (Figure 2B). Then, expressions of Cyclin B1, Bcl-2 were decreased, caspase9 (Figure 2B), caspase3 activity (Figure 2C) were increased. Inversely, PFTα reduced p53 expression (Figure 2B). These results indicated that the combination of metformin and 2DG resumed p53 function through suppressing expressions of MDM2 and MDM4 leading to G2-M arrest and apoptosis in MCF-7 and MCF/Dox cells. Then, we asked whether resumed p53 function increased cytotoxicity of doxorubicin in MCF-7/Dox cells. IC50 value of doxorubicin was 9.35±1.30μM in the presence of AB143228, which was significantly lower compare with doxorubicin alone (21.12±1.89μM) (Figure 2D). These results indicated that the combination of metformin and 2DG increased cytotoxicity of doxorubicin in MCF-7/Dox cells by resuming p53 function.

### Combination of metformin and 2DG enhanced doxorubicin accumulation in MCF-7/Dox cells

**Figure 3:**
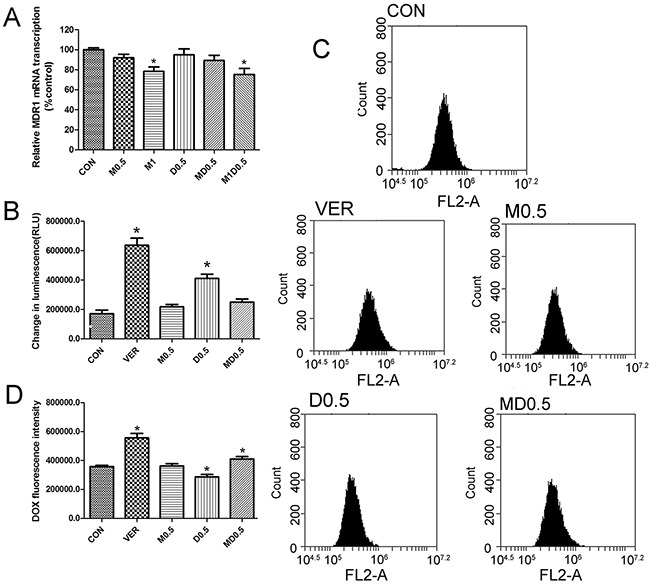
Metformin and 2-deoxyglucose(2DG) combination increased doxorubicin accumulation in MCF-7/Dox cells A. mRNA expression of MDR1 was tested by quantitative real-time PCR analysis(the control group is set at 1). Verapamil(10μM) was positive group in B, C and D. (B) P-gp ATP enzyme activity was tested by Pgp-Glo™ Assay Systems. Metformin(0.5mM) plus 2DG(0.5mM) had no effect on P-gp mRNA expression and P-gp ATP enzyme activity. (C) Doxorubicin accumulation was determined by flow cytometric. (D) DOX fluorescence intensity analysis of C. Columns, data are expressed as mean ± SD. *P < 0.05 Significantly different from control group. M0.5: 0.5 mM metformin, D0.5: 0.5 mM 2DG, MD0.5: 0.5 mM metformin plus 0.5 mM 2DG, M1D0.5: 1mM metformin plus 0.5 mM 2DG.

Metformin inhibited MDR1 expression of MCF-7/Dox cells by blocking MDR1 gene transcription. In order to confirm the result, real-time PCR was performed to detect the changes in MDR1 mRNA upon treatment with metformin and 2DG. 1mM metformin significantly decreased MDR1 mRNA expression. But results of 0.5mM metformin and 0.5mM 2DG had no statistical differences (Figure 3A). Next, we detected P-gp ATPase upon treatment with metformin or 2DG. Verapamil was used as positive control. Verapamil and 2DG significantly increased P-gp ATPase activity, but the combination of metformin and 2DG had no effect on P-gp ATPase activity (Figure 3B). Finally, doxorubicin accumulation assay was performed to detect the changes in P-gp function (Figure 3C). Verapamil, which was positive control, significantly increased doxorubicin accumulation. Also, the combination of metformin and 2DG increased doxorubicin accumulation in MCF-7/Dox cells (Figure 3D). Since combination of metformin and 2DG had no effect on MDR1 mRNA expression and P-gp ATPase activity, we asked whether combination of two drugs inhibited energy metabolism leading to P-gp function suppression.

### Combination of metformin and 2DG hampered metabolism in MCF-7 and MCF-7/Dox cells

**Figure 4:**
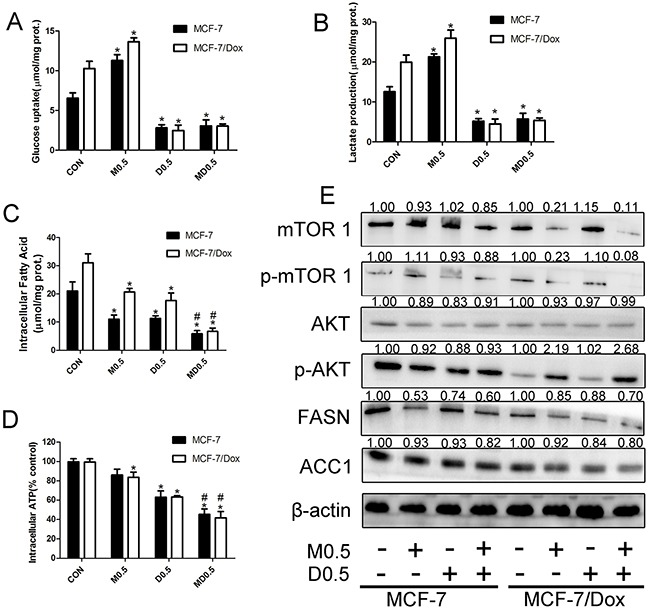
The inhibitory effect of metformin plus 2-deoxyglucose(2DG) on energy metabolism Cells were treated with indicated drugs for 24h. A, B, C, D. Combination of metformin and 2DG inhibited glucose uptake, lactate, fatty acid and ATP production in both cell lines. E. Metformin plus 2DG increased p-AKT expression and down-regulated mTOR and p- mTOR, as well as FASN and ACC1 expression. Columns, data are expressed as mean ± SD. *P < 0.05 Significantly different from control group. M0.5: 0.5 mM metformin, D0.5: 0.5 mM 2DG, MD0.5: 0.5 mM metformin plus 0.5 mM 2DG.

Metformin strongly increased glucose uptake and lactate production but 2DG suppressed glycolysis and prevented metformin-induced glucose uptake and lactate production in both cell lines (Figure 4A–4B). Combination of the two drugs significantly reduced fatty acid production (Figure 4C). Thus, ATP production significantly reduced upon treatment with combination of metformin and 2DG (Figure 4D). Meanwhile, we detected expressions of mTOR, AKT(two important metabolic regulation protein) FASN and ACC1. Metformin alone, combination of metformin and 2DG markedly decreased the expressions of mTOR and p-mTOR and increased p-AKT expression in MCF-7/Dox cells. Combination of the two drugs significantly reduced expressions of FASN and ACC1 (Figure 4E). These results indicated that the metabolism was significantly suppressed upon treatment with metformin plus 2DG. The combination of metformin and 2DG initiated a strong metabolic stress in MCF-7 and MCF-7/Dox cells.

### Doxorubicin selectively increased the induced effect of metformin plus 2DG in MCF-7/Dox cells

**Figure 5:**
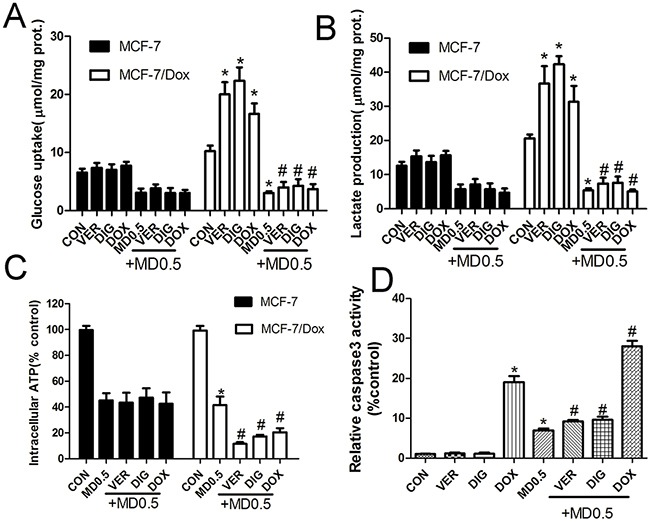
Interaction effects between P-gp substrate and combination of metformin and 2-deoxyglucose(2DG) on energy metabolism A, B, C. Glucose uptake, lactate and ATP production was determined after the addition of indicated drugs for 24h. D. Caspase3 activity was determined by flow cytometry. Columns, data are expressed as mean ± SD. *P < 0.05 Significantly different from control group. VER: 10 μM verapamil, DIG: 10 μM digoxin, DOX: 10 μM doxorubici, M0.5: 0.5 mM metformin, D0.5: 0.5 mM 2DG, MD0.5: 0.5 mM metformin plus 0.5 mM 2DG.

Since P-gp requires 2 ATPs to export one substrate molecule, we asked whether doxorubicin increased the induced effect of metformin plus 2DG. Verapamil and digoxin(two classic P-gp substrates) were used as positive control. Incubation with 10μM verapamil, 10μM digoxin and 10μM doxorubicin for 24h selectively increased glucose uptake and lactate produce in MCF-7/Dox cells. The combination of metformin and 2DG strongly suppressed increased glucose uptake and lactate produce induced by P-gp substrates (Figure 5A–5B). Meanwhile, verapamil, digoxin and doxorubicin selectively aggravated the ATP depletion in MCF-7/Dox cells (Figure 5C). Finally, verapamil, digoxin and doxorubicin increased the caspase3 activity induced by metformin plus 2DG (Figure 5D). These results indicated that doxorubicin selectively aggravated ATP depletion leading to increasing apoptosis in MCF-7/Dox cells.

## DISCUSSION

Based on the On the Origin of Species by Means of Natural(Charles Darwin), the natural selection leads to evolutionary change of species over time [20]. Nowell hypothesised that natural selection occurs in the evolutionary change of tumours and drug resistance possibly [21]. In the presence of cytotoxic drugs, cancer cells evolved to be drug resistance cells to adapt to the disadvantageous environment. This hypothesis is entirely reasonable. Resistance to anticancer drugs is a complex process [7]. Compared with MCF-7 cells, MDR1 (Figure 3A), MRP1(Multidrug Resistance associated Protein 1)(data not shown), p53, MDM2 and MDM4 were over-expressed (Figure 2B); glucose uptake was higher (Figure 4A) in MCF-7/Dox cells.

Metformin or 2-deoxyglucose(2DG) exhibits strong antiproliferative action or sensitizes cancer cells to the action of radiation in numerous cancer cell lines [22–25]. Futhermore, metformin reverses MDR through down-regulating P-gp expression and 2DG reverses MDR through suppressing metabolism in MCF-7/adr cells [26, 27]. Combination of metformin and 2DG selectively increased cytotoxicity of doxorubicin in MCF-7/Dox cells. That means they have potential to reverse MDR (Figure 1C–1D).

Overexpression of ABC transporters was the most studied MDR mechanism. Theoretically, suppressing P-gp function is the most efficient way to reverse P-gp-mediated MDR. There are three general approaches to inhibit P-gp function: blocking its drug-pump function, inhibiting its expression and inhibiting P-gp ATPase activity [16, 28]. Combination of 0.5mM metformin and 0.5mM 2DG increased doxorubicin accumulation in MCF-7/Dox cells (Figure 3C–3D). Increased doxorubicin accumulation means that P-gp function was suppressed. Interestingly, 0.5mM metformin plus 0.5mM 2DG had no effect on MDR1 mRNA expression and P-gp ATPase activity (Figure 3A–3B). Our previous study showed that P-gp could not mediate the efflux transport of metformin [18]. We hypothesised that inhibiting energy metabolism was a candidate mechanism to suppressing P-gp function based on ABC transporters hydrolyze ATP to transport substrates. Combination of metformin and 2DG significantly suppress energy metabolism and reduced ATP production (Figure 5A–5D). Our results showed that inhibiting energy metabolism maybe effective to suppress P-gp function.

p53 responses to several cellular stresses including DNA damage, hypoxia and oncogene activation [29]. Overexpression of MDM2, thereby reducing p53 levels, was the major mechanism to indirectly suppress p53 function [30]. Recent studies shown that p53 was closely linked with drug chemosensitivity and drug resistance [31]. Here, we showed that expressions of MDM2 and MDM4 were significantly increased in MCF-7/Dox cells (Figure 2B). Trino, S. et al. shown that Nutlin-3a (small-molecule MDM2 antagonist) treatment reduced viability and induced p53-mediated apoptosis in acute lymphoblastic leukemia cells [32]. Combination of metformin and 2DG intensively reduced expressions of MDM2 and MDM4 (Figure 2B), leaded G2-M arrest and apoptosis (Figure 2A–2B)in MCF-7/Dox cells. The same results showed by p53 activator AB143228 (Figure 2B). Overexpression of p53 increases chemosensitivity in multidrug-resistant osteosarcoma cell lines [13]. AB143228 selectively increased cytotoxicity of doxorubicin in MCF-7/Dox cells (Figure 2D). Therefore, combination of the two drugs increased chemosensitivity in MCF-7/Dox cells by resuming p53 function.

Plentiful literatures have reported that disrupting cancer cell metabolism was effect strategy to fight cancer [33, 34]. Furthermore, dual inhibition of tumor energy induced by metformin plus 2DG had been proved effective to fight cancer in several cancer cell lines [35, 36, 18]. Combination of metformin and 2DG significantly reduced energy metabolism in MCF-7 and MCF-7/Dox cells (Figure 4A–4D). Metformin had already been proved a AMP-activated protein kinase(AMPK) activator in numerous cells including MCF-7 and MCF-7/Dox cell [26, 37]. AMPK is a key sensor of cellular energy and the negative regulator of mammalian target of rapamycin(mTOR). mTOR integrates the presence of growth factors, energy levels, glucose and amino acids to modulate metabolic status and cellular responses. mTOR inhibition means the nutrient supply is low and metabolism is suppressed [38, 15]. Combination of metformin and 2DG markedly decreased the expression of mTOR and p-mTOR (Figure 4E). AKT, a regulator of glycolysis, stimulates glycolysis by increasing the expression and translocation of glucose transporters [15, 39]. Combination of metformin and 2DG obviously increased p-AKT expression in MCF-7/Dox cell. (Figure 4E). Meanwhile, Combination of the two drugs reduced acetyl-CoA carboxylase 1(ACC1) and fatty acid synthase(FASN) (two AMPK downstream proteins) expression (Figure 4E). These results further indicated that energry metabolism was significantly suppressed upon treatment with metformin plus 2DG. A non-toxic substrate can consume the resources of resistant cells and reduce their capacity for proliferation and invasion [17]. Our previous study showed that P-gp substrate selectively increased the induced effect of combination of metformin and 2DG on apoptosis in K562/Dox cells [18]. Combination of metformin and 2DG strongly suppressed increased energy requirement by P-gp substrate (Figure 5A–5B). Meanwhile, verapamil, digoxin and doxorubicin selectively aggravated the ATP depletion (Figure 5C) in MCF-7/Dox cells (Figure 5D). These results indicated that doxorubicin selectively aggravated ATP depletion leading to increasing cell apoptosis in MCF-7/Dox cells.

Based on all above results, combination of metformin and 2-deoxyglucose inhibits the overexpression of MDM2 and MDM4, resumes p53 function, initiates strong metabolic stress and enhances doxorubicin accumulation in MCF-7/Dox cells. Furthermore, doxorubicin selectively aggravates metabolic stress induced by metformin plus 2DG in MCF-7/Dox cells. The mutually reinforcing effect made the combination of metformin and 2DG had a better effect on reversing MDR. Our observations highlight the importance of the combination of metformin and 2DG in reversing MDR of MCF-7/Dox cells.

## MATERIALS AND METHODS

### Cell lines and culture conditions

The human breast cancer cell lines MCF-7 and MCF-7/Dox cells were purchased from KeyGen Biotech Co., Ltd.(Nanjing China) and cultured in RPMI 1640 supplemented with 10% fetal bovine serum (FBS) and 100 units/mL penicillin, 100 mg/mL streptomycin at 37°C and 5% CO_2_. MCF-7/Dox cells were cultured in medium with 2 mg/mL doxorubicin and grown in doxorubicin-free culture medium for more than 2 weeks before assay.

### Chemicals

Metformin, 2-deoxyglucose, doxorubicin, vera-pamil, digoxin, MTT were all purchased from Dalian Meilun Biotech Co., Ltd.(China). p53 activator AB143228 was purchased from Abcam Company. p53 inhibitor PFTα was obtained from Selleck Chemicals LLC.

### Antibodies

Western blot analysis was performed with antibodies against p-mTOR, p-AKT(Cell Signaling Technology), p53, MDM2, MDM4, Cyclin B1, Cyclin D1, BAX, Bcl-2, Caspase 9, mTOR, AKT, FASN, ACC1, β-actin(Proteintech Group).

### MTT assay

MCF-7 and MCF-7/Dox cells(5×10^3^/well) were seeded in 96-well plates. 24h later, cells were incubated with indicated drugs. MTT assay was performed as described previously. Absorbance was measured at 570nm using a microplate reader(Bio-Rad, USA). Percentage cell viability was calculated based on the absorbance of the drug-treated cells relative to the absorbance of the control vehicle-treated cells.

### Cell cycle assay

To identify cell cycle, 5×10^5^/well MCF-7 and MCF-7/Dox cells were seeded in 6-well plates and treated with indicated drugs for 24h. Cells were detected by FxCycle™ PI/Rnase Staining (Thermo Fisher Scientific) and flow cytometric analysis(FACScalibar, BD).

### Western blot

MCF-7 and MCF-7/Dox cells were seeded in 6-well plates and treated with indicated drugs for 24h. Cell lysis and immunoblotting were performed as previously described [18]. β-actin was used as loading control. The protein bands were detected by ChemiDoc^TM^ XRS+ Imaging system(Bio-Rad). Quantification of protein expression was analyzed through Image Lab™ Software(Bio-Rad).

### Caspase3 assay

MCF-7 and MCF-7/Dox cells were seeded in 6-well plates and treated with indicated drugs for 24h. Caspase 3 activity was detected by Caspase 3 Colorimetric Assay Kit (KeyGen Biotech) according to the manufacturer's instructions. Absorbance was measured at 405nm using a microplate reader (Bio-Rad, USA). Percentage caspase 3 activity was calculated based on the absorbance of the drug-treated cells relative to the absorbance of the control vehicle-treated cells.

### Quantitative real-time PCR

MCF-7 and MCF-7/Dox cells were seeded in 6-well plates and treated with indicated drugs for 24h. Total RNA was isolated with an RNA isolation kit(Takara, Japan). cDNA was generated from 1μg of total RNA using PrimeScript® RT Reagent Kit (Takara Biotechnology) and was amplified using SYBR® Premix Ex Taq™ Kit(Takara Biotechnology) by ABI PRISM® 7500 Real-Time PCR System(Applied Biosystems) as previously described [19]. β-actin was used as normalization control. Primers used were as follows: 5’-GGAGCCTACTTGGTGGCACATAA-3’ and 5’-TGGCATAGTCAGGAGCAAATGAAC-3’ for MDR1, and 5’-ATTGAACACGGCATTGTCAC-3’ and 5’-CATCGGAACCGCTCATTG-3’ for β-actin. The fold change for MDR1 relative to the control was calculated using the comparative ΔΔCt method.

### P-gp ATPase assay

P-gp ATPase assay was detected by Pgp-Glo™ Assay Systems(Promega Biotech Co., Ltd.) according to the manufacturer's instructions. Na_3_VO_4_ is a selective inhibitor of P-gp, samples treated with Na_3_VO_4_ have no P-gp ATPase activity. The difference in luminescent signal between Na_3_VO_4_-treated samples and test compound-treated samples represents P-gp ATPase activity in the presence of the test compound. Verapamil was used as positive control.

### Doxorubicin accumulation assay

MCF-7/Dox cells were seeded in 6-well plates and pretreated with indicated drugs for 24h. Verapamil was used as positive control. After pretreatment, the cells were incubated with 20μM doxorubicin in PBS at 37°C for 45min. Then, cells were collected and washed with ice-cold PBS twice and suspended in 0.5mL of PBS. The fluorescence intensity of doxorubicin was determined by flow cytometric analysis(FACScalibur, BD).

### Fatty acid, glucose, lactate and ATPassays

MCF-7 and MCF-7/Dox cells were seeded in 6-well plates and cultured in non-phenol red RPMI 1640(Solarbio, Life Sciences) for 24h. Then, cells were incubated with drugs as indicated. 24h later, fatty acid, glucose, lactate and ATP were detected by Fatty Acid Determination Kit, Glucose Determination Kit, Lactate Determination Kit(KeyGen Biotech) and ATP Assay Kit(Beyotime Instiute of Biotechnology) respectively according to the manufacturer's instructions.

### Statistical analysis

All data were expressed as means ± SD. All experiments were repeated at least three times. Non-paired t test was used to estimate the statistical differences between two groups. One-way analysis of varianve(ANOVA) was used to determine the differences between three or more groups. All analyses were carried out using GraphPad Prism 5.0. The value of P < 0.05 was considered to be statistically significant.

## Abbreviations

P-glycoprotein: P-gp
adenosine triphosphate: ATP
multidrug resistance: MDR
multidrug resistance gene 1: MDR1
murine doubleminute 2: MDM2
murine doubleminute 4: MDM4
AMP-activated protein kinase: AMPK
mammalian target of rapamycin: mTOR
protein kinase B: AKT
acetyl-CoA carboxylase 1: ACC1
fatty acid synthase: FASN

## References

[1] Torre LA, Bray F, Siegel RL, Ferlay J, Lortet-Tieulent J, Jemal A Global cancer statistics, 2012. CA Cancer J Clin.

[2] Murray S, Briasoulis E, Linardou H, Bafaloukos D, Papadimitriou C Taxane resistance in breast cancer: mechanisms, predictive biomarkers and circumvention strategies. Cancer Treat Rev.

[3] Amiri-Kordestani L, Basseville A, Kurdziel K, Fojo AT, Bates SE Targeting MDR in breast and lung cancer: discriminating its potential importance from the failure of drug resistance reversal studies. Drug Resist Updat.

[4] Kathawala RJ, Gupta P, Ashby CR, Chen Z The modulation of ABC transporter-mediated multidrug resistance in cancer: A review of the past decade. Drug Resist Updat.

[5] Gillet JP, Gottesman MM Mechanisms of multidrug resistance in cancer. Methods Mol Biol.

[6] Silva R, Vilas-Boas V, Carmo H, Dinis-Oliveira RJ, Carvalho F, M de Lourdes Bastos, Remiao F Modulation of P-glycoprotein efflux pump: induction and activation as a therapeutic strategy. Pharmacol Ther.

[7] Gottesman MM, Lavi O, Hall MD, Gillet JP Toward a Better Understanding of the Complexity of Cancer Drug Resistance. Annu Rev Pharmacol Toxicol.

[8] Deben C, Deschoolmeester V, Lardon F, Rolfo C, Pauwels P TP53 and MDM2 genetic alterations in non-small cell lung cancer: Evaluating their prognostic and predictive value. Crit Rev Oncol Hematol.

[9] Soussi T, Wiman KG TP53: an oncogene in disguise. Cell Death Differ.

[10] Zhou W, Tian D, He J, Wang Y, Zhang L, Cui L, Jia L, Zhang L, Li L, Shu Y, Yu S, Zhao J, Yuan X (2016). Repeated PM2.5 exposure inhibits BEAS-2B cell P53 expression through ROS-Akt-DNMT3B pathway-mediated promoter hypermethylation. Oncotarget.

[11] Tal P, Eizenberger S, Cohen E, Goldfinger N, Pietrokovski S, Oren M, Rotter V (2016). Cancer therapeutic approach based on conformational stabilization of mutant p53 protein by small peptides. Oncotarget.

[12] I Ben Sahra, Laurent K, Giuliano S, Larbret F, Ponzio G, Gounon P, Le Marchand-Brustel Y, Giorgetti-Peraldi S, Cormont M, Bertolotto C, Deckert M, Auberger P, Tanti JF et al.Targeting cancer cell metabolism: the combination of metformin and 2-deoxyglucose induces p53-dependent apoptosis in prostate cancer cells. Cancer Res.

[13] Ye S, Shen J, Choy E, Yang C, Mankin H, Hornicek F, Duan Z p53 overexpression increases chemosensitivity in multidrug-resistant osteosarcoma cell lines. Cancer Chemother Pharmacol.

[14] Warburg O On the origin of cancer cells. Science.

[15] Bost F, Decoux-Poullot AG, Tanti JF, Clavel S Energy disruptors: rising stars in anticancer therapy?. Oncogenesis.

[16] Gottesman MM, Fojo T, Bates SE Multidrug resistance in cancer: role of ATP-dependent transporters. Nat Rev Cancer.

[17] Kam Y, Das T, Tian H, Foroutan P, Ruiz E, Martinez G, Minton S, Gillies RJ, Gatenby RA Sweat but no gain: inhibiting proliferation of multidrug resistant cancer cells with “ersatzdroges”. Int J Cancer.

[18] Xue C, Wang C, Liu Q, Meng Q, Sun H, Huo X, Ma X, Liu Z, Ma X, Peng J, Liu K Targeting P-glycoprotein expression and cancer cell energy metabolism: combination of metformin and 2-deoxyglucose reverses the multidrug resistance of K562/Dox cells to doxorubicin. Tumour Biol.

[19] Chen T, Wang C, Liu Q, Meng Q, Sun H, Huo X, Sun P, Peng J, Liu Z, Yang X, Liu K Dasatinib reverses the multidrug resistance of breast cancer MCF-7 cells to doxorubicin by downregulating P-gp expression via inhibiting the activation of ERK signaling pathway. Cancer Biol Ther.

[20] Gerlinger M, Swanton C How Darwinian models inform therapeutic failure initiated by clonal heterogeneity in cancer medicine. Br J Cancer.

[21] Nowell PC The clonal evolution of tumor cell populations. Science.

[22] DePeralta DK, Wei L, Ghoshal S, Schmidt B, Lauwers GY, Lanuti M, Chung RT, Tanabe KK, Fuchs BC (2016). Metformin prevents hepatocellular carcinoma development by suppressing hepatic progenitor cell activation in a rat model of cirrhosis. Cancer.

[23] Shen C, Ka SO, Kim SJ, Kim JH, Park BH, Park JH Metformin and AICAR regulate NANOG expression via the JNK pathway in HepG2 cells independently of AMPK. Tumour Biol.

[24] Kobayashi H, Nishimura H, Matsumoto K, Yoshida M Identification of the determinants of 2-deoxyglucose sensitivity in cancer cells by shRNA library screening. Biochem Biophys Res Commun.

[25] Huang CC, Wang SY, Lin LL, Wang PW, Chen TY, Hsu WM, Lin TK, Liou CW, Chuang JH Glycolytic inhibitor 2-deoxyglucose simultaneously targets cancer and endothelial cells to suppress neuroblastoma growth in mice. Dis Model Mech.

[26] Kim HG, Hien TT, Han EH, Hwang YP, Choi JH, Kang KW, Kwon KI, Kim BH, Kim SK, Song GY, Jeong TC, Jeong HG Metformin inhibits P-glycoprotein expression via the NF-kappaB pathway and CRE transcriptional activity through AMPK activation. Br J Pharmacol.

[27] Kaplan O, Navon G, Lyon RC, Faustino PJ, Straka EJ, Cohen JS Effects of 2-deoxyglucose on drug-sensitive and drug-resistant human breast cancer cells: toxicity and magnetic resonance spectroscopy studies of metabolism. Cancer Res.

[28] Chufan EE, Kapoor K, Ambudkar SV Drug-protein hydrogen bonds govern the inhibition of the ATP hydrolysis of the multidrug transporter P-glycoprotein. Biochem Pharmacol.

[29] Turner N, Moretti E, Siclari O, Migliaccio I, Santarpia L, D’Incalci M, Piccolo S, Veronesi A, Zambelli A, G Del Sal, Di Leo A Targeting triple negative breast cancer: is p53 the answer?. Cancer Treat Rev.

[30] Moll UM, Petrenko O The MDM2-p53 interaction. Mol Cancer Res.

[31] Duffy MJ, Synnott NC, McGowan PM, Crown J, O’Connor D, Gallagher WM p53 as a target for the treatment of cancer. Cancer Treat Rev.

[32] Trino S, Iacobucci I, Erriquez D, Laurenzana I, Luca L, Ferrari A, Rora AG, Papayannidis C, Derenzini E, Simonetti G, Lonetti A, Venturi C, Cattina F (2016). Targeting the p53-MDM2 interaction by the small-molecule MDM2 antagonist Nutlin-3a: a new challenged target therapy in adult Philadelphia positive acute lymphoblastic leukemia patients. Oncotarget.

[33] Ledoux S, Yang R, Friedlander G, Laouari D Glucose depletion enhances P-glycoprotein expression in hepatoma cells: role of endoplasmic reticulum stress response. Cancer Res.

[34] Harper ME, Antoniou A, Villalobos-Menuey E, Russo A, Trauger R, Vendemelio M, George A, Bartholomew R, Carlo D, Shaikh A, Kupperman J, Newell EW, Bespalov IA Characterization of a novel metabolic strategy used by drug-resistant tumor cells. FASEB J.

[35] Cheong JH, Park ES, Liang J, Dennison JB, Tsavachidou D, Nguyen-Charles C, K Wa Cheng, Hall H, Zhang D, Lu Y, Ravoori M, Kundra V, Ajani J Dual inhibition of tumor energy pathway by 2-deoxyglucose and metformin is effective against a broad spectrum of preclinical cancer models. Mol Cancer Ther.

[36] I Ben Sahra, Tanti JF, Bost F The combination of metformin and 2-deoxyglucose inhibits autophagy and induces AMPK-dependent apoptosis in prostate cancer cells. Autophagy.

[37] Morales DR, Morris AD Metformin in cancer treatment and prevention.Annu. Rev Med.

[38] Rebsamen M, Pochini L, Stasyk T, de Araujo ME, Galluccio M, Kandasamy RK, Snijder B, Fauster A, Rudashevskaya EL, Bruckner M, Scorzoni S, Filipek PA, Huber KV SLC38A9 is a component of the lysosomal amino acid sensing machinery that controls mTORC1. Nature.

[39] Elstrom RL, Bauer DE, Buzzai M, Karnauskas R, Harris MH, Plas DR, Zhuang H, Cinalli RM, Alavi A, Rudin CM, Thompson CB Akt stimulates aerobic glycolysis in cancer cells. Cancer Res.

